# Supplementary material for: Differences in Cerebral Glucose Metabolism in ALS Patients with and without C9orf72 and SOD1 Mutations
Source: Cells. 2023 Mar 18;12(6):933. doi: 10.3390/cells12060933 (PMC10047407; doi:10.3390/cells12060933)
Supplement: Supplementary file 1 [file cells-12-00933-s001.zip › cells-2228473-SM.pdf]

Supplementary Materials

Table S1: Global Cluster peak coordinates variants and statistics of relative glucose metabolism comparing *C9orf72*-ALS with *SOD1*-ALS.

Table S2: Global Cluster peak coordinates variants and statistics of relative hypo- and hypermetabolism when comparing groups of ALS patients (i.e., $^{\mathit{SOD1}\text{-matched}}$sALS, $^{\mathit{C9orf72}\text{-matched}}$sALS, *SOD1*-ALS, and *C9orf72*-ALS) to healthy controls.

Table S3: Volume-of-interest (VOI)-based Mann–Whitney U tests of ^{18}F-FDG SUVR between healthy controls and *SOD1*-ALS (n = 22) and matched sporadic ALS ($^{\mathit{SOD1}\text{-matched}}$sALS; n = 21).

Table S4: Volume-of-interest (VOI)-based Mann–Whitney U tests of ^{18}F-FDG SUVR between healthy controls and *C9orf72*-ALS (n = 48) and matched sporadic ALS ($^{\mathit{C9orf72}\text{-matched}}$sALS; n = 48).

Table S5: Global Cluster peak coordinates variants and statistics of relative glucose metabolism comparing *C9orf72*-ALS with $^{\mathit{C9orf72}\text{-matched}}$sALS.

Table S6: Clinical and genetic data of ALS patients with a *SOD1* gene mutation.

Figure S1: T-statistical map detailing patterns of relative hypometabolism (yellow) and hypermetabolism (blue) (A) in *C9orf72*-ALS, when compared to healthy controls, and (B) in $^{\mathit{C9orf72}\text{-matched}}$sALS, when compared to healthy controls.

Figure S2: T-statistical map detailing patterns of relative hypometabolism (yellow) and hypermetabolism (blue) (A) in *SOD1*-ALS, when compared to healthy controls, and (B) in $^{\mathit{SOD1}\text{-matched}}$sALS, when compared to healthy controls.

Figure S3: Regional relative glucose metabolism in key volume-of-interest regions in four ALS groups.

Table S1: Global Cluster peak coordinates variants and statistics of relative glucose metabolism comparing *C9orf72*-ALS with *SOD1*-ALS, while correcting for age, sex, scanner type, and King's disease stage. L = left; R = right, SUVR = standardized uptake value ratio.

**Cluster level**		**Voxel level**					**Anatomical region cluster**
$p_{FWE-corr}$	K_{ext}	$p_{FWE-corr}$	**T-value**	**Peak voxel coordinate** X	Y	Z	
**Relative hypometabolism in *C9orf72*-ALS vs. sporadic ALS**							
<0.001	1020	0.171	4.59	-26	-12	10	Lentiform nucleus (L)
		0.227	4.49	-22	18	-14	Frontal orbital cortex (L)
		0.627	4.01	-26	10	0	Lentiform nucleus (L)
		0.739	3.90	-16	0	-6	Lentiform nucleus (L)
		0.842	3.77	-22	-8	-14	Amygdala (L)
<0.001	1687	0.173	4.59	20	-70	-40	Cerebellum
		0.295	4.38	-16	-70	-40	Cerebellum
		0.552	4.09	4	-60	-36	Cerebellum
		0.764	3.87	38	-62	-44	Cerebellum
		0.947	3.59	2	-64	-46	Cerebellum
<0.001	1330	0.324	4.34	30	0	6	Lentiform nucleus (R)
		0.637	4.00	-4	-24	-26	Brainstem (pons)
		0.733	3.90	26	-14	-12	Amygdala (R)
		0.743	3.89	28	16	-8	Insular cortex (R)
		0.753	3.88	26	20	-10	Frontal orbital cortex (R)
		0.823	3.80	28	-16	0	Lentiform nucleus (R)
		0.843	3.77	22	0	-2	Lentiform nucleus (R)
		0.976	3.49	38	2	-8	Insular cortex (R)
		0.983	3.45	14	-22	-24	Brainstem (midbrain)

Table S2: Global Cluster peak coordinates variants and statistics of relative hypo- and hypermetabolism when comparing groups of ALS patients (i.e., $^{SOD1\text{-matched}}$sALS, $^{C9orf72\text{-matched}}$sALS, *SOD1*-ALS, and *C9orf72*-ALS) to healthy controls.

SPM condition (contrast)	p_{height} uncorr	Cluster level		t-value	mm			Brain area
		p_{FWE}	equiv$_k$		x	y	z	
**Clusters of relative hypometabolism when compared to healthy controls**								
$^{SOD1\text{-matched}}$sALS	<0.001	<0.001	10113	6.52	-32	60	-4	Frontal pole; Inferior and Middle Frontal gyrus; Frontal operculum cortex; Subcallosal Cortex; Insula; Putamen.
		<0.001	1787	5.69	10	-62	30	Cingulate gyrus (posterior division); Precuneus.
		0.001	690	5.41	-6	-18	-14	Ventral diencephalon, Thalamus.
		0.004	565	5.03	58	-62	24	Lateral occipital cortex; Angular gyrus.
		0.003	583	4.88	-56	-50	-22	Inferior and Middle temporal gyrus .
		0.009	468	4.61	-54	-62	36	Lateral occipital cortex.
$^{C9orf72\text{-matched}}$sALS	<0.001	<0.001	37102	8.01	-20	62	0	Frontal pole; Frontal opercular cortex; Orbitofrontal cortex; Precuneus.
		<0.001	4797	7.41	-58	-52	-18	Inferior and Middle temporal gyrus; Lateral occipital cortex; Angular gyrus.
		0.036	380	4.71	20	18	66	Superior frontal gyrus.
SOD1-ALS	<0.001	<0.001	4589	7.96	-30	60	-4	Frontal pole; Precentral gyrus; Inferior and Middle frontal gyrus; Frontal operculum cortex; Insular cortex.
		0.041	317	6.48	24	-32	78	Postcentral gyrus; Superior parietal lobule.
		<0.001	1374	6.12	-44	-60	56	Lateral occipital cortex; Angular gyrus.
		<0.001	2023	6.00	10	-62	32	Cingulate gyrus (posterior division); Precuneus; Intracalcarine cortex.
		<0.001	5950	5.92	34	62	-6	Frontal pole; Inferior and Middle frontal gyrus; Precentral gyrus; Frontal orbital cortex; Subcallosal Cortex; Postcentral gyrus.
		<0.001	1170	5.91	58	-54	40	Angular gyrus; Lateral occipital cortex; Supramarginal gyrus.
		0.002	660	5.06	-56	-60	-18	Angular gyrus; Inferior temporal gyrus; Lateral occipital cortex; Inferior and Middle temporal gyrus.
		0.010	458	4.73	22	6	-6	Putamen.
		0.013	431	4.72	54	-22	12	Planum temporale.

		0.006	514	4.34	-16	-16	12	Thalamus.
C9orf72-ALS	<0.001	<0.001	50787	8.29	-32	52	10	Frontal pole; Frontal operculum cortex; Inferior frontal gyrus; Middle frontal gyrus; Thalamus; Cingulate gyrus posterior division; Precuneus,
		0.001	883	5.41	-68	-30	-6	Inferior and Middle temporal gyrus; Lateral occipital cortex.
**Clusters of relative hypermetabolism when compared to healthy controls**								
SOD1-matched sALS	<0.001	0.047	305	4.67	16	-94	-2	Cerebellum.
C9orf72-matched sALS	<0.001	<0.001	2578	5.63	0	-66	-46	Cerebellum.
C9orf72-ALS	<0.001	<0.001	9047	7.33	2	-66	-46	Cerebellum and Medulla.
		0.001	815	6.71	-44	-4	-24	Medial temporal lobe (hippocampus, amygdala).
		0.001	798	6.09	30	-20	-14	Medial temporal lobe (hippocampus); insular cortex.
		0.024	426	5.46	26	42	-4	Frontal orbital cortex; Central opercular cortex; Insular cortex.
		0.044	358	4.47	20	-88	4	Occipital pole.
SOD1-ALS	<0.001	<0.001	1637	5.25	2	-54	-46	Cerebellum.

Table S3: Volume-of-interest (VOI)-based t-tests of ^{18}F-FDG SUVR between healthy controls and *SOD1*-ALS (n = 22) and matched sporadic ALS ($^{SOD1\text{-matched}}$sALS; n = 21). Significant *p*-values, after applying FDR correction (α^{FDR} = 0.05), are shown in bold. IQR, interquartile range; Md, median; sALS = sporadic ALS; SOD1-ALS = SOD1-associated ALS; SUVR = standardized uptake value ratio.

Md (IQR)	CON	*SOD1*-ALS	Test statistic	p_{FDR} value	$^{SOD1\text{-}matched}$sALS	Test statistic	p_{FDR} value
Mid Frontal gyrus	1.135 (0.07)	1.100 (0.07)	122.0	**0.040**	1.090 (0.03)	81.5	0.005
Precentral gyrus	1.010 (0.04)	1.015 (0.04)	175.0	0.347	1.000 (0.04)	191.0	0.715
Straight gyrus	1.020 (0.04)	0.975 (0.05)	90.0	**0.005**	0.990 (0.06)	113.5	**0.037**
Orbitofrontal cortex	1.070 (0.06)	1.000 (0.07)	61.0	**0.002**	1.000 (0.06)	57.0	**0.001**
Inferior frontal gyrus	1.100 (0.05)	1.075 (0.06)	66.5	**0.002**	1.050 (0.06)	39.5	**2.96E^{-4}**
Superior frontal gyrus	1.015 (0.04)	1.010 (0.05)	189.0	0.471	1.010 (0.05)	195.0	0.778
Medial orbital gyrus	1.005 (0.05)	0.960 (0.05)	77.0	**0.003**	0.960 (0.05)	89.5	**0.012**
Lateral orbital gyrus	1.075 (0.06)	1.025 (0.05)	70.0	**0.002**	1.010 (0.06)	58.5	**9.12E^{-4}**
Posterior orbital gyrus	0.975 (0.04)	0.940 (0.03)	104.0	**0.014**	0.960 (0.05)	133.0	0.094
Subgenual frontal cortex	0.860 (0.05)	0.830 (0.05)	132.5	0.067	0.830 (0.05)	122.5	0.058
Subcallosal area	0.830 (0.08)	0.825 (0.05)	175.0	0.337	0.820 (0.07)	170.5	0.430
Pre-subgenual frontal cortex	1.050 (0.06)	1.030 (0.06)	111.0	**0.025**	1.040 (0.07)	158.5	0.273
Hippocampus	0.745 (0.07)	0.770 (0.18)	165.0	0.265	0.770 (0.06)	150.5	0.202
Amygdala	0.690 (0.05)	0.720 (0.07)	146.0	0.125	0.730 (0.05)	117.5	**0.043**
Anterior temporal lobe (med)	0.740 (0.04)	0.740 (0.04)	185.5	0.427	0.750 (0.05)	143.5	0.158
Anterior temporal lobe (lateral)	0.863 (0.03)	0.860 (0.06)	213.5	0.893	0.870 (0.05)	200.5	0.849
Parahippocampal and ambient gyri	0.790 (0.04)	0.780 (0.03)	181.0	0.397	0.780 (0.05)	183.5	0.580
Superior temporal gyrus, posterior	0.990 (0.05)	1.010 (0.08)	183.5	0.425	1.010 (0.04)	147.5	0.187
Middle and inferior temporal gyrus	0.950 (0.04)	0.940 (0.04)	168.5	0.296	0.950 (0.06)	177.5	0.540
Fusiform gyrus	0.845 (0.05)	0.840 (0.04)	216.0	0.919	0.830 (0.05)	182.5	0.581
Posterior temporal lobe	1.020 (0.03)	1.000 (0.03)	164.5	0.264	1.020 (0.03)	181.5	0.577
Superior temporal gyrus, anterior	0.815 (0.06)	0.840 (0.05)	174.0	0.347	0.830 (0.06)	136.5	0.111
Postcentral gyrus	1.010 (0.04)	1.010 (0.03)	184.5	0.426	1.010 (0.04)	202.5	0.866
Superior parietal gyrus	1.075 (0.05)	1.090 (0.05)	176.0	0.338	1.070 (0.06)	196.0	0.777
Inferolateral remainder of the parietal lobe	1.075 (0.04)	1.055 (0.04)	146.5	0.121	1.050 (0.03)	98.0	**0.016**

Lateral remainder of the occipital lobe	1.060 (0.05)	1.100 (0.05)	87.0	**0.005**	1.100 (0.07)	110.0	**0.033**
Lingual gyrus	1.135 (0.08)	1.180 (0.06)	121.0	**0.040**	1.190 (0.07)	124.5	0.062
Cuneus	1.215 (0.08)	1.220 (0.07)	155.5	0.181	1.210 (0.07)	204.0	0.875
Thalamus	0.995 (0.07)	0.935 (0.06)	114.5	**0.030**	0.940 (0.07)	111.5	**0.034**
Insula	0.940 (0.06)	0.930 (0.04)	150.0	0.141	0.930 (0.05)	156.5	0.259
Cingulate gyrus, anterior	1.020 (0.06)	1.005 (0.06)	173.0	0.346	1.000 (0.09)	170.0	0.437
Cingulate gyrus, posterior	1.190 (0.07)	1.160 (0.05)	142.0	0.113	1.140 (0.07)	127.5	0.072
Cerebellum	0.905 (0.06)	0.960 (0.09)	124.0	**0.043**	0.980 (0.06)	68.0	**0.002**
Medulla	0.695 (0.11)	0.715 (0.10)	200.5	0.659	0.740 (0.09)	148.5	0.190
Midbrain	0.770 (0.06)	0.740 (0.09)	132.0	0.069	0.760 (0.06)	177.5	0.522
Pons	0.605 (0.05)	0.625 (0.06)	143.5	0.115	0.650 (0.05)	100.5	**0.016**
Lentiform nucleus	1.175 (0.06)	1.080 (0.07)	79.0	0.003	1.100 (0.08)	96.5	**0.016**

Table S4: Volume-of-interest (VOI)-based t-tests of ^{18}F-FDG SUVR between healthy controls and *C9orf72*-ALS (n = 48) and matched sporadic ALS ($^{C9orf72\text{-matched}}$sALS; n = 48). Significant *p*-values, after applying FDR correction (α^{FDR} = 0.05), are shown in bold. *C9orf72*-ALS, *C9orf72*-associated ALS; IQR, interquartile range; Md, median; sALS= sporadic ALS; SUVR = standardized uptake value ratio.

Md (IQR)	CON	*C9orf72*-ALS	Test statistic	p_{FDR} value	$^{C9orf72\text{-matched}}$sALS	Test statistic	p_{FDR} value
Mid Frontal gyrus	1.135 (0.07)	1.070 (0.07)	151.0	**4.76E-5**	1.085 (0.06)	174.0	**1.85E-4**
Precentral gyrus	1.010 (0.04)	0.990 (0.05)	321.0	0.054	1.010 (0.04)	458.0	0.810
Straight gyrus	1.020 (0.04)	0.970 (0.06)	204.0	**7.22E-4**	0.950 (0.08)	156.0	**8.02E-5**
Orbitofrontal cortex	1.070 (0.06)	1.010 (0.06)	139.5	**3.70E-5**	1.000 (0.07)	86.0	**3.99E-6**
Inferior frontal gyrus	1.100 (0.04)	1.050 (0.06)	73.5	**1.49E-6**	1.055 (0.06)	88.5	**2.24E-6**
Superior frontal gyrus	1.015 (0.05)	0.990 (0.04)	310.0	**0.041**	1.000 (0.06)	377.5	0.227
Medial orbital gyrus	1.005 (0.05)	0.970 (0.07)	219.0	**0.001**	0.960 (0.04)	104.0	**4.37E-6**
Lateral orbital gyrus	1.075 (0.06)	1.020 (0.06)	138.0	**3.70E-5**	1.000 (0.07)	111.5	**6.16E-6**
Posterior orbital gyrus	0.975 (0.04)	0.960 (0.06)	322.5	0.053	0.945 (0.06)	272.0	**0.014**
Subgenual frontal cortex	0.860 (0.05)	0.835 (0.08)	370.0	0.189	0.820 (0.08)	286.5	**0.021**
Subcallosal area	0.830 (0.08)	0.800 (0.10)	323.0	0.052	0.810 (0.10)	324.5	0.063
Pre-subgenual frontal cortex	1.050 (0.06)	1.005 (0.12)	311.0	**0.041**	1.015 (0.12)	317.0	0.052
Hippocampus	0.745 (0.07)	0.780 (0.08)	336.5	0.078	0.770 (0.09)	380.5	0.238
Amygdala	0.690 (0.05)	0.745 (0.07)	216.5	**0.001**	0.720 (0.07)	309.5	**0.041**
Anterior temporal lobe (med)	0.740 (0.04)	0.755 (0.04)	276.0	**0.014**	0.730 (0.06)	471.0	0.928
Anterior temporal lobe (lateral)	0.865 (0.03)	0.870 (0.04)	470.5	0.898	0.860 (0.05)	424.0	0.536
Parahippocampal and ambient gyri	0.790 (0.04)	0.790 (0.03)	464.5	0.857	0.790 (0.05)	433.5	0.593
Superior temporal gyrus, posterior	0.990 (0.05)	1.000 (0.06)	440.0	0.660	1.015 (0.06)	371.5	0.204
Middle and inferior temporal gyrus	0.950 (0.04)	0.940 (0.04)	377.5	0.211	0.930 (0.03)	283.5	**0.020**
Fusiform gyrus	0.845 (0.05)	0.840 (0.06)	443.0	0.652	0.830 (0.05)	394.5	0.316
Posterior temporal lobe	1.020 (0.03)	1.010 (0.03)	421.0	0.504	1.010 (0.04)	403.0	0.364
Superior temporal gyrus, anterior	0.815 (0.06)	0.820 (0.05)	441.0	0.651	0.810 (0.08)	451.0	0.756
Postcentral gyrus	1.010 (0.04)	0.980 (0.04)	309.0	**0.041**	1.010 (0.05)	432.5	0.602
Superior parietal gyrus	1.075 (0.05)	1.070 (0.04)	424.0	0.519	1.085 (0.04)	371.0	0.213
Inferolateral remainder of the parietal lobe	1.075 (0.04)	1.040 (0.04)	213.0	**0.001**	1.050 (0.03)	278.5	**0.016**

Lateral remainder of the occipital lobe	1.060 (0.05)	1.120 (0.07)	198.5	**6.04E^{-4}**	1.100 (0.06)	233.0	**0.004**
Lingual gyrus	1.135 (0.08)	1.190 (0.12)	250.5	**0.005**	1.190 (0.11)	254.0	**0.007**
Cuneus	1.215 (0.08)	1.255 (0.12)	304.0	**0.037**	1.230 (0.09)	326.0	0.064
Thalamus	0.995 (0.07)	0.880 (0.10)	130.5	**2.47E^{-5}**	0.930 (0.09)	242.5	**0.004**
Insula	0.940 (0.06)	0.925 (0.03)	349.0	0.108	0.920 (0.06)	294.5	**0.026**
Cingulate gyrus, anterior	1.020 (0.06)	0.975 (0.08)	293.0	**0.026**	1.005 (0.09)	370.5	0.207
Cingulate gyrus, posterior	1.190 (0.10)	1.120 (0.05)	213.0	**9.74E^{-4}**	1.150 (0.05)	339.0	0.092
Cerebellum	0.905 (0.06)	1.025 (0.07)	91.0	**2.92E^{-6}**	0.985 (0.07)	153.5	**8.14E^{-5}**
Medulla	0.695 (0.11)	0.790 (0.07)	148.0	**4.32E^{-5}**	0.740 (0.07)	304.5	**0.037**
Midbrain	0.770 (0.06)	0.810 (0.08)	372.0	0.192	0.790 (0.07)	474.5	0.941
Pons	0.605 (0.05)	0.680 (0.08)	179.5	**2.36E^{-4}**	0.650 (0.07)	224.0	**0.003**
Lentiform nucleus	1.175 (0.06)	1.155 (0.08)	410.5	0.430	1.110 (0.09)	260.5	**0.009**

Table S5: Global Cluster peak coordinates variants and statistics of relative glucose metabolism comparing *C9orf72*-ALS with $^{C9orf72\text{-matched}}$sALS. L = left; R = right, SUVR = standardized uptake value ratio.

Cluster level		Voxel level					Anatomical region cluster
$p_{FWE-corr}$	K_{ext}	$p_{FWE-corr}$	T-value	Peak voxel coordinate X	Y	Z	
**Relative hypometabolism in *C9orf72*-ALS vs. sporadic ALS**							
0.038	380	0.003	5.67	16	-30	2	Thalamus (R).
		0.961	3.48	8	-10	2	Thalamus (R).
0.001	843	0.383	4.17	2	-24	52	Precentral gyrus (medial segment).
		0.727	3.83	8	-18	64	Precentral gyrus.
		0.928	3.57	6	-46	68	Precuneus.
**Relative hypermetabolism in *C9orf72*-ALS vs. sporadic ALS**							
0.012	511	0.089	4.68	-6	-34	-54	Medulla.
		0.184	4.45	10	-26	-44	Medulla.

Table S6: Clinical and genetic data of ALS patients with an *SOD1* gene mutation.

Subject	Disease onset	UMN involvement	Variant
SOD 01	Spinal	Bulbar; upper and lower limbs	G94C
SOD 02	Spinal	Upper and lower limbs	E50K
SOD 03	Spinal	Bulbar; upper and lower limbs	G94C
SOD 04	Spinal	Bulbar; upper and lower limbs	D91A, I114T
SOD 05	Spinal	Upper and lower limbs	D91A
SOD 06	Spinal	Upper limbs	I152T
SOD 07	Bulbar	Upper and lower limbs	I114T
SOD 08	Thoracic/respiratory	Thoracic, upper and lower limbs	G139V
SOD 09	Spinal	Bulbar; upper limbs	D91A
SOD 10	Spinal	Upper and lower limbs	G94C
SOD 11	Spinal	Upper and lower limbs	N140D
SOD 12	Spinal	Bulbar; upper and lower limbs	D91A
SOD 13	Spinal	Upper limbs	G94C
SOD 14	Bulbar	Bulbar; upper and lower limbs	D91A
SOD 15	Bulbar	Upper and lower limbs	D91A
SOD 16	Spinal	Upper and lower limbs	D91A
SOD 17	Spinal	Upper and lower limbs	D91A
SOD 18	Spinal	Upper and lower limbs	G94C
SOD 19	Spinal	Bulbar; upper and lower limbs	D91A
SOD 20	Spinal	Upper and lower limbs	D91A
SOD 21	Spinal	Bulbar; upper and lower limbs	L39V
SOD 22	Spinal	Upper limbs	G94C

Appendix G: Figure S1

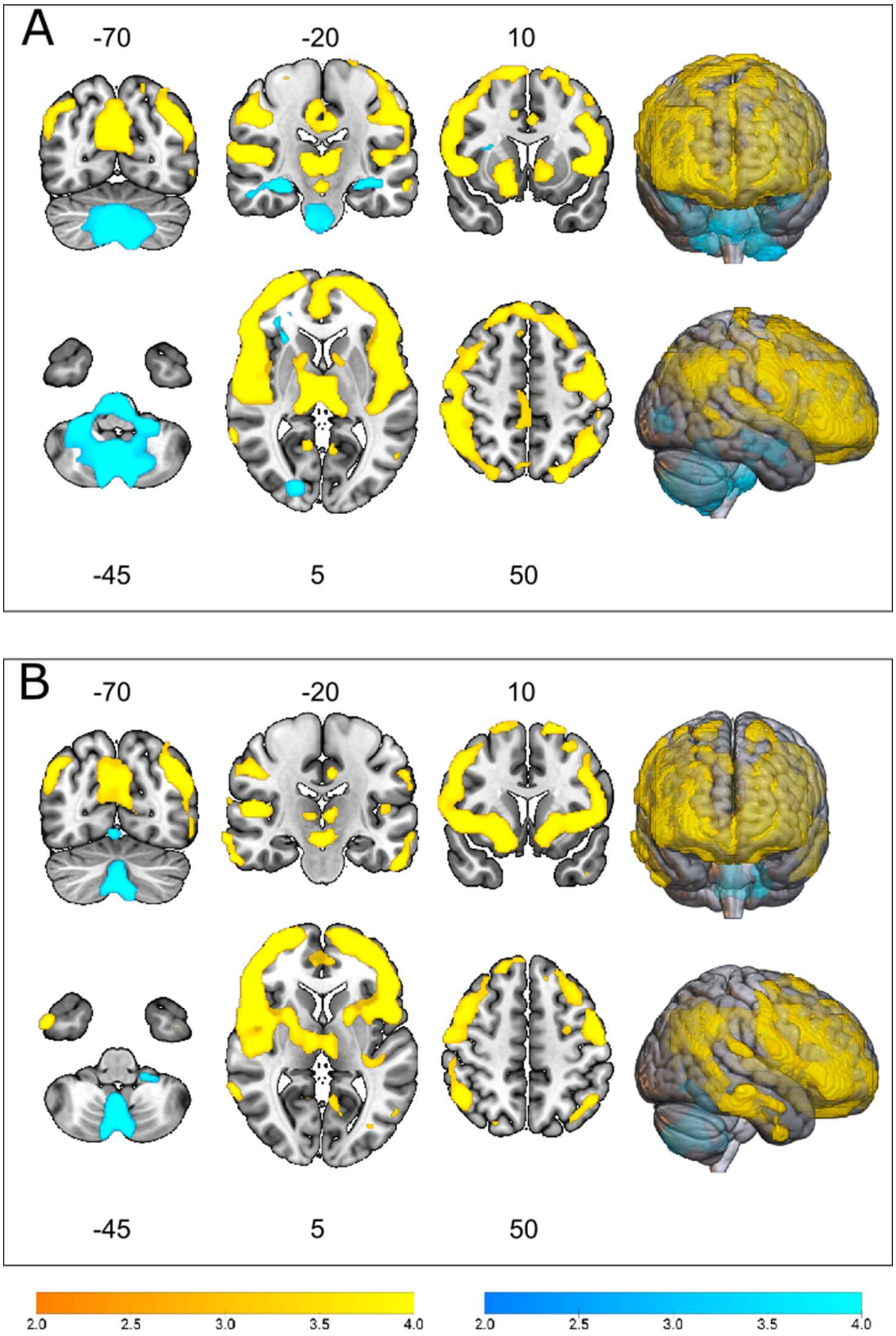

Figure S1. T-statistical map detailing patterns of relative hypometabolism (yellow) and hypermetabolism (blue) (A) in *C9orf72*-ALS, when compared to healthy controls, and (B) in $^{C9orf72\text{-matched}}$sALS, when compared to healthy controls. Analyses are thresholded at $p_{uncorr} < 0.001$ at voxel level and $p_{FWE} < 0.05$ at cluster level and are corrected for age at FDG , sex, and scanner type. Clusters are overlaid on a T1 template.

Appendix H: Figure S2

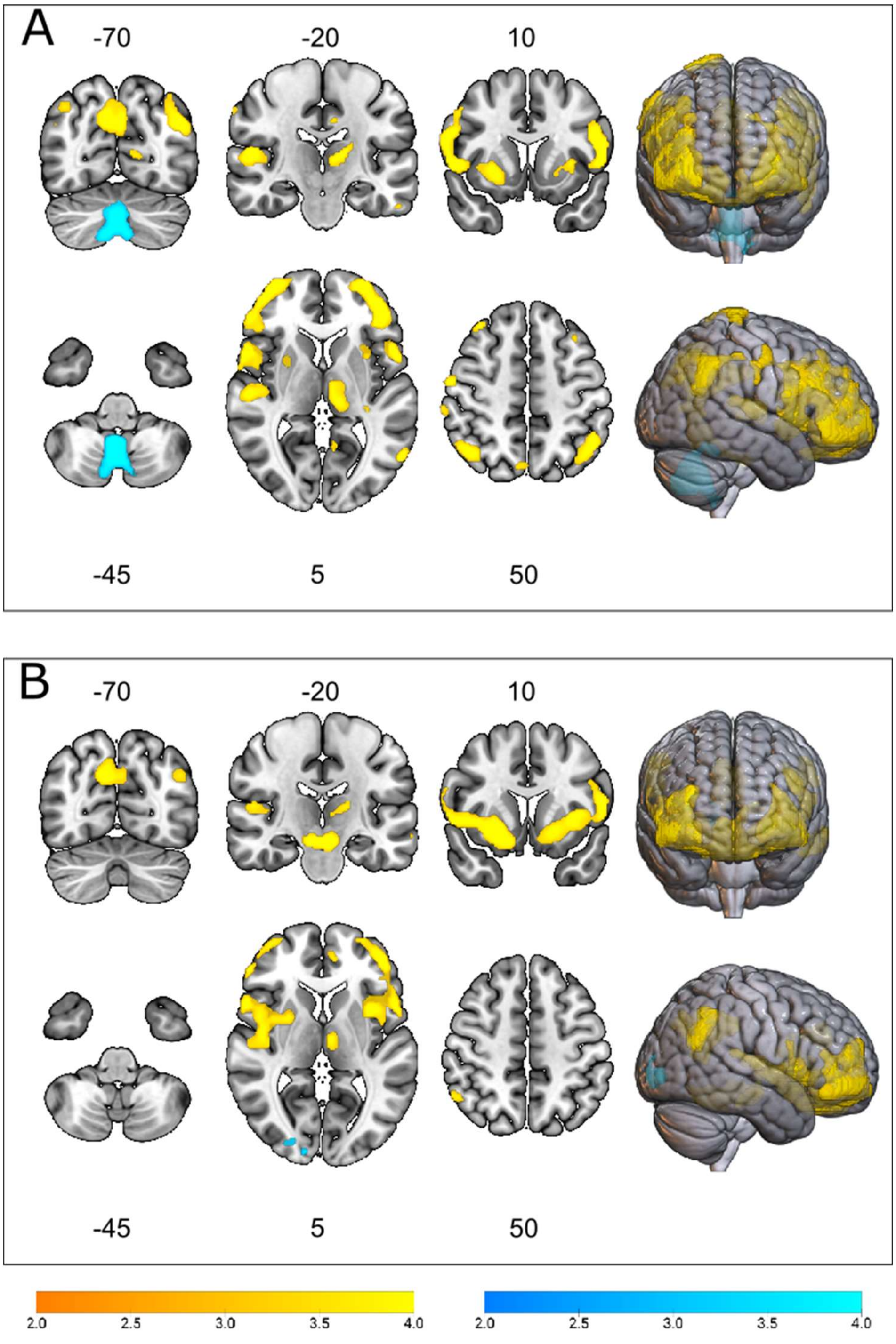

Figure S2. T-statistical map detailing patterns of relative hypometabolism (yellow) and hypermetabolism (blue) (A) in *SOD1*-ALS, when compared to healthy controls, and (B) in $^{SOD1\text{-}matched}$sALS, when compared to healthy controls. Analyses are thresholded at $p_{uncorr} < 0.001$ at voxel level and $p_{FWE} < 0.05$ at cluster level and are corrected for age at FDG PET, sex, and scanner type. Clusters are overlaid on a T1 template.

Appendix I: Figure S3

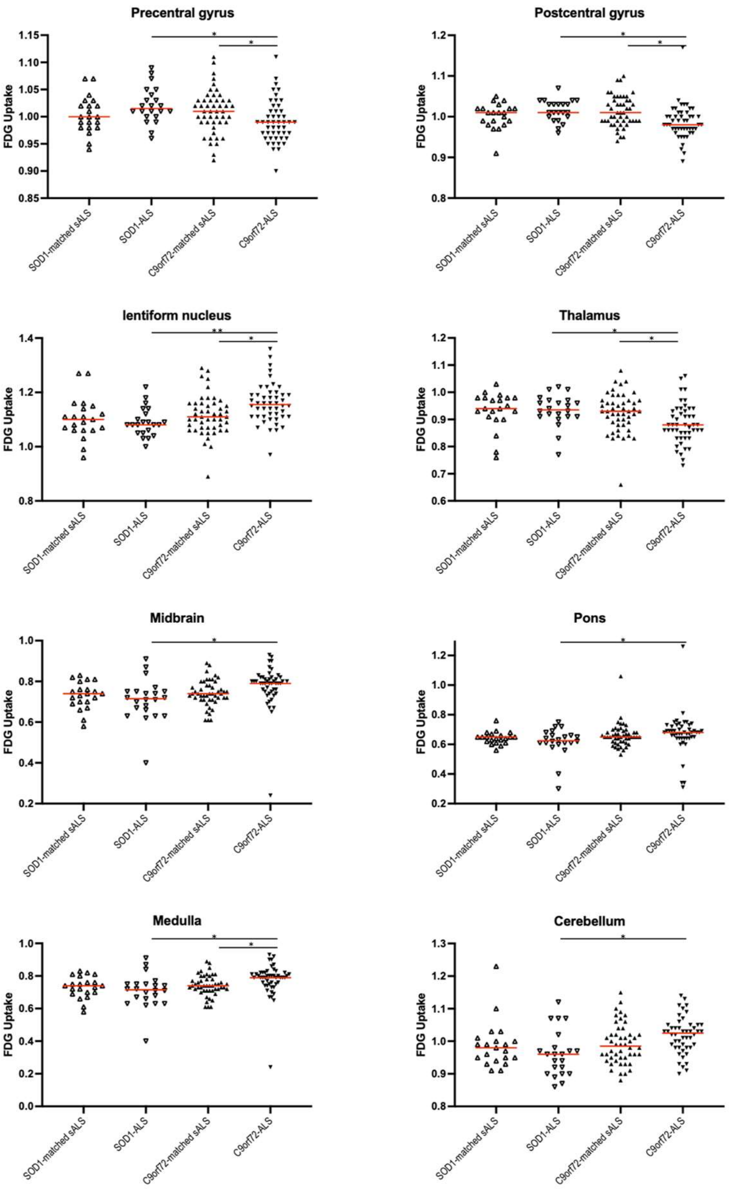

Figure S3. Regional relative glucose metabolism in key volume-of-interest regions (i.e., precentral gyrus, postcentral gyrus, lentiform nucleus, thalamus, midbrain, pons, medulla, cerebellum) in four ALS groups (i.e., $^{C9orf72\text{-matched}}$sALS, $^{SOD1\text{-matched}}$sALS, *SOD1*-ALS, *C9orf72*-ALS). Significance is denoted as: *$P_{FDR} < 0.05$, **$P_{FDR} < 0.005$.
